# Diagnostic value of MRI combined with ultrasound for lymph node metastasis in breast cancer: Protocol for a meta-analysis

**DOI:** 10.1097/MD.0000000000016528

**Published:** 2019-07-26

**Authors:** Dechun Cai, Tong Lin, Kailin Jiang, Zhizhong Sun

**Affiliations:** a The First Affiliated Hospital of Guangzhou University of Chinese Medicine; b Guangzhou University of Chinese Medicine, Guangzhou, China.

**Keywords:** breast cancer, diagnostic, lymph node, metastasis, MRI, ultrasound

## Abstract

**Background:**

Early diagnosis and treatment of breast cancer are important to prevent fatal tumor progression. Axillary lymph node (ALN) status is the most significant prognostic factor for diagnosing overall survival in breast cancer patients. Axillary lymph node dissection (ALND) is regarded as the reference standard for determining ALN status. However, ALND is an invasive therapy with high morbidity and complications such as lymphedema, seroma and nerve injury. Comparatively, magnetic resonance imaging (MRI) and ultrasound are noninvasive and non-radiative techniques that are common imaging methods to diagnose breast cancer lymph node metastasis. Many studies have investigated the diagnostic value of MRI combined with ultrasound for breast cancer ALN metastasis, but the evidence has been insufficient to apply these modalities when diagnosing new patients.

**Methods:**

We will search electronic databases including PubMed, EMbase, The Cochrane Library, Chinese Biomedical Database, WangFang Database, and China National Knowledge Infrastructure. The language of studies is limited in English or Chinese. The final search includes articles published in June, 2018. Stata 14.0 software will be used for all statistical analyses, and Quality Assessment of Diagnostic Accuracy Studies (QUADAS-2) will be utilized to evaluate the quality of the included studies. Meta-regression and subgroup analyses will be performed to explore heterogeneity, which will be derived from the different countries of origin of the included studies. Deeks’ funnel plot asymmetry test will be demonstrated the inexistence of publication bias.

**Result:**

This study will provide a rational synthesis of current evidences for magnetic resonance imaging combined with ultrasound for breast cancer.

**Conclusion:**

The conclusion of this study will provide evidence for the diagnostic value of MRI combined with ultrasound for lymph node metastasis in breast cancer.

**Registration:**

PROS-PERO CRD42019134474

## 1 Introduction

Breast cancer is the most common tumor type in women, the most frequent cause of cancer-related death in female patients and the fifth most common cause of cancer-related deaths in all patients.^[1–5]^ Worldwide, the reported mortality rate of breast cancer was 12.9% in 2012.^[1]^ Early diagnosis and treatment of breast cancer are important to prevent fatal tumor progression.^[2,6,7]^ Additionally, it is essential to identify metastasis to determine whether adjuvant systemic chemotherapy or postoperative radiation should be performed. Axillary lymph node (ALN) status is the most significant prognostic factor for diagnosing overall survival in breast cancer patients.^[8]^ Axillary lymph node dissection (ALND) is regarded as the reference standard for determining ALN status, as its diagnostic accuracy is almost 100%, and it is now a part of the conventional basic mastectomy for local disease control.^[8–13]^ However, ALND is an invasive therapy with high morbidity and complications such as lymphedema, seroma and nerve injury.^[14]^ Comparatively, magnetic resonance imaging (MRI) and ultrasound are noninvasive and non-radiative techniques that are common imaging methods to diagnose breast cancer lymph node metastasis.^[15,16]^ Many studies have investigated the diagnostic value of MRI combined with ultrasound for breast cancer ALN metastasis, but the evidence has been insufficient to apply these modalities when diagnosing new patients. Therefore, we performed a meta-analysis to summarize these studies and more fully assess the diagnostic value of MRI combined with ultrasound for diagnosing breast cancer lymph node metastasis.

## 2 Methods

### 2.1 Ethics committee or institutional review board

Not applicable. It is a meta-analysis, not a clinical trial.

### 2.2 Study registration

We have been registered the protocol on the International Prospective Register of Systematic Reviews (PROSPERO) (registration number, CRD42019134474).

### 2.3 Literature search

We will search electronic databases, including Web of Science, PubMed, EMbase, The Cochrane Library, Chinese Biomedical Database, Wan-Fang Database, and China National Knowledge Infrastructure. The final search included articles published in August 2018. Both free words and MeSH terms will be adopted, and the search strategy will be as follows: (“MR” OR “magnetic resonance imaging” OR “diffusion magnetic resonance imaging”) AND (“ultrasound imaging” OR “ultrasonic tomograph” OR “ultrasonography”) AND (“breast neoplasms” OR “breast tumors” OR “breast cancer”) AND (“lymph node”) AND (“metastases”).

### 2.4 Study selection

Two reviewers will independently evaluate the eligible literature. Discrepancies between reviewers will be resolved through discussion and consultation with a third author. The following inclusion criteria will be applied:

(1) diagnostic trials with 2-by-2 tabulated data including true positive (TP), false positive (FP), true negative (TN) and false negative (FN) rates that could be extracted directly or indirectly;
(2) breast cancer diagnoses will be confirmed by histopathological examination, including intraoperative sections, sentinel lymph node biopsy (SLNB) and ALND; and
(3) patients will be suspected to have axillary or cervical lymph-node metastases, and all patients underwent both magnetic resonance (MR) and ultrasound examination.

There was no limit on patient age, race or nationality, but the number of cases needed to be greater than 25. The exclusion criteria will be as follows:

(1) case reports, reviews, and seminar articles;
(2) patients will be not examined by both MR and ultrasound; and
(3) duplicate publications.

### 2.5 Data extraction

The following data will be extracted using a self-made form:

(1) general study characteristics consisting of the first author's name, publishing data, sample size, blinding method, ultrasound frequency, field of MRI, and research type;
(2) diagnostic values including sensitivity, specificity, TP, FP, FN, TN, and area under curve (AUC).

Where there are missing data of included studies in data extraction, we attempted to contact the original authors for detailed information through email or telephone. If we failed to acquire sufficient data, we would only analyze the available data and perform an intention-to-treat analysis to address the potential impacts of the missing data on results in discussion section.

### 2.6 Quality assessment

Two reviewers will independently evaluate all included studies based on the revised Quality Assessment of Diagnostic Accuracy Studies (QUADAS-2) checklist. Each item in the QUADAS-2 will be marked with “yes”, “no” or “unclear”, representing low risk, high risk and unclear, respectively.

### 2.7 Statistical analysis

The pooled data, including sensitivity, specificity, positive likelihood ratio (+LR), negative likelihood ratio (−LR), diagnosis odds ratios (DOR) and their 95% confidence intervals (95% CIs), will be evaluated to assess diagnostic performance. Furthermore, Spearman's correlation analysis will be performed to examine a threshold effect. An inverse correlation will be demonstrated among the sensitivity and specificity if there is a threshold effect. The *Q* test and *I*^2^ statistic will be applied to evaluate the heterogeneity of sensitivity and specificity among the studies. Notable heterogeneity will be suggested by a *P* value less than .1 for the *χ*^2^ test and an *I*^2^ value larger than 50%, and accordingly, the random-effect model will be employed. Otherwise the fixed effect model will be applied. Univariate meta-regression and subgroup analysis to explore potential sources of heterogeneity will be performed. Deeks’ funnel plot will be developed to evaluate publication bias. All statistical analyses will be performed using Stata 14.0 software.

## 3 Discussion

Breast cancer is the most commonly diagnosed cancer and the leading cause of cancer-related death in female patients.^[17,18]^ Over the past few decades, improvements in the diagnosis and treatment of early-staged breast cancer have proven to significantly reduce its morbidity and mortality.^[19]^ Breast cancer patients with early-stage symptoms often cannot be clearly clinically diagnosed. Usually, a definitive diagnosis occurs 2 to 3 years after disease onset.^[20]^ Moreover, many patients develop lymph node metastasis, resulting from the phenotype of breast cancer cells, which are loosely connected and easily detached.^[21]^ Lymph node metastasis and tumor progression pose a serious threat to a patient's overall survival. Therefore, it is critical to determine the presence of lymph node metastasis in breast cancer patients during surgery.^[22]^ Recently, a number of methods have been applied to accurately diagnose lymph node metastasis, including SLNB and ALND,^[23]^ both of which are invasive tests that are easily complicated by wound infection, upper extremity lymphedema or cacesthesia.

MRI and ultrasound are noninvasive and non-radiative techniques that are common imaging methods to diagnose breast cancer lymph node metastasis. MRI has a high sensitivity for detecting additional lesions that cannot be detected by ultrasound or mammography.^[24]^ Studies have reported that MRI has high resolution of soft tissue and achieves semi-quantitative analysis of the tissue lesion by measuring apparent diffusion coefficient values.^[25]^ It is reported that MRI has a higher value of diagnosing lymph node metastasis in breast cancer when compared with PET/CT. Ultrasound imaging has advantages of multi-angle real-time scanning and evaluating tissue flow. A retrospective study revealed that the sensitivity, specificity and overall accuracy of ultrasonography in diagnosing breast cancer lymph node metastasis were 69.4%, 81.8%, and 77.0%, respectively. Therefore, the diagnostic value of ultrasound in lymph node metastasis detection has been proven.^[19]^ However, current ultrasound technology cannot detect changes in the internal structure of lymph nodes that are generated by metastasis.^[26]^ In these cases, MRI combined with ultrasound can lead to a more precise diagnosis.

A previous study described that MRI combined with ultrasound showed moderate sensitivity and specificity in predicting the status of lymph node metastasis in breast cancer patients. However, these techniques remain insufficient in sensitivity and specificity to exclude the use of sentinel lymph node biopsy and axillary dissection.^[27]^ Several studies have focused on one of the imaging techniques, MRI or ultrasound, while several other studies have investigated the combination of MRI and ultrasound. Notably, a meta-analysis has not been conducted to directly explore the combination of MRI and breast ultrasonography as a diagnostic method for lymph node metastasis in breast cancer.

Therefore, the aim of this study is to assess the value of magnetic resonance imaging combined with ultrasound for detecting lymph node metastasis in breast cancer patients.

## Author contributions

**Conceptualization:** Dechun Cai.

**Data curation:** Tong Lin.

**Formal analysis:** Kailin Jiang.

**Methodology:** Zhizhong Sun.
